# Secondary Metabolites with α-Glucosidase Inhibitory Activity from the Mangrove Fungus *Mycosphaerella* sp. SYSU-DZG01

**DOI:** 10.3390/md17080483

**Published:** 2019-08-20

**Authors:** Pei Qiu, Zhaoming Liu, Yan Chen, Runlin Cai, Guangying Chen, Zhigang She

**Affiliations:** 1 School of Chemistry, Sun Yat-Sen University, Guangzhou 510275, China; 2 State Key Laboratory of Applied Microbiology Southern China, Guangdong Institute of Microbiology, Guangdong Academy of Sciences, Guangzhou 510070, China; 3 Key Laboratory of Tropical Medicinal Plant Chemistry of Ministry of Education, Hainan Normal University, Haikou 571158, China; 4 South China Sea Bio-Resource Exploitation and Utilization Collaborative Innovation Center, Guangzhou 510006, China

**Keywords:** secondary metabolites, *Mycosphaerella* sp., asperchalasine, α-glucosidase

## Abstract

Four new metabolites, asperchalasine I (**1**), dibefurin B (**2**) and two epicoccine derivatives (**3** and **4**), together with seven known compounds (**5**–**11**) were isolated from a mangrove fungus *Mycosphaerella* sp. SYSU-DZG01. The structures of compounds **1**–**4** were established from extensive spectroscopic data and HRESIMS analysis. The absolute configuration of **1** was deduced by comparison of ECD data with that of a known structure. The stereostructures of **2**–**4** were further confirmed by single-crystal X-ray diffraction. Compounds **1**, **8** and **9** exhibited significant α-glucosidase inhibitory activity with IC_50_ values of 17.1, 26.7 and 15.7 μM, respectively. Compounds **1**, **4**, **6** and **8** showed antioxidant activity by scavenging DPPH· with EC_50_ values ranging from 16.3 to 85.8 μM.

## 1. Introduction

Diabetes mellitus (DM), a chronic metabolic disorder disease, is caused by the lack of insulin secretion (type І diabetes mellitus) or insufficient insulin sensitivity (type ІІ diabetes mellitus) [1,2], and the typical characteristic of the latter is post-prandial hyperglycemia. α-Glucosidase is a kind of membrane-bounded enzyme which is mainly found in intestinal epithelium cells and leads to the increase of blood glucose levels by hydrolyzing the glycosidic bonds of a polysaccharide [3,4,5]. As a result of that, α-Glucosidase inhibitors (AGIs), such as acarbose, miglitol and voglibose have become a widespread medical treatment in type ІІ diabetes mellitus according to their glycemic control ability [6,7]. Nevertheless, existing AGIs often cause many side effects including abdominal pain, flatulence, diarrhea and other gastrointestinal disorders [8,9]. Hence, many natural medicine chemists were attracted to develop α-glucosidase inhibitors with lower toxicity and side effects for potential use. Some new α-glucosidase inhibitors have been researched like flavipesolides A–C [10], asperteretal E [11] and so on [12,13], and the discovery of better α-glucosidase inhibitors is still an urgent need.

**Figure 1:**
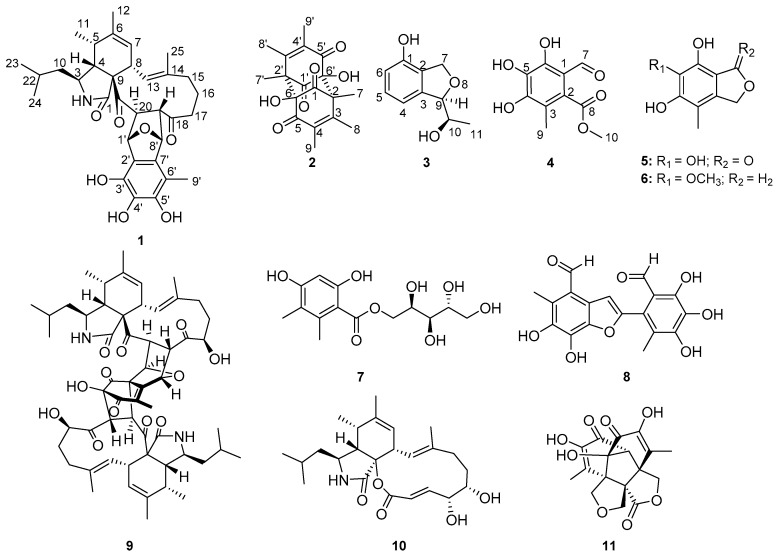
Chemical structures of **1**–**11**.

Marine fungi are proved to be rich sources of structurally unique and bioactive secondary metabolites. *Mycosphaerella* sp., which contributes the largest genus of Ascomycota, is a common plant pathogen widely distributed in terrestrial plant and marine environment [14,15,16]. As part of our ongoing investigation on new secondary metabolites from marine fungi in the South China Sea [17,18,19,20,21], a mangrove fungus, *Mycosphaerella* sp. SYSU-DZG01, collected from the fruit of the mangrove plant *Bruguiera*, Hainan Dongzhai Harbor Mangrove Reserve attracted our attention because the EtOAc extract of the solid fermentation medium exhibited significant α-glucosidase inhibitory activity. Chemical investigation of the bioactive extract (Figure 1) lead to the discovery of four new metabolites, asperchalasine I (**1**), dibefurin B (**2**) and two epicoccine derivatives, (*R*)-9-((*R*)-10-hydroxyethyl)-7,9-dihydroisobenzofuran-1-ol (**3**), 2-methoxycarbonyl-4,5,6-trihydroxy-3-methyl-benzaldehyde (**4**), together with seven known compounds, epicoccone B (**5**) [22], 1,3-dihydro-5-methoxy-7-methylisobenzofuran (**6**) [23], paeciloside A (**7**) [24], epicoccolide B (**8**) [25], asperchalasine A (**9**) [26], aspochalasin I (**10**) [27] and epicolactone (**11**) [28]. Their structures were established by extensive spectroscopic data and single-crystal X-ray diffraction analysis. Asperchalasine I possesses a distinct T-shaped skeleton containing one epicoccine moiety and one cytochalasan moiety. In bioactivity assays, compounds **1**, **8** and **9** exhibited α-glucosidase inhibitory activity and **1**, **4**, **6** and **8** showed antioxidant activity by scavenging DPPH·. Herein, the isolation, structure elucidation, α-glucosidase inhibitory and antioxidant activities of these compounds are reported.

## 2. Results

### 2.1. Structure Elucidation

**Figure 2:**
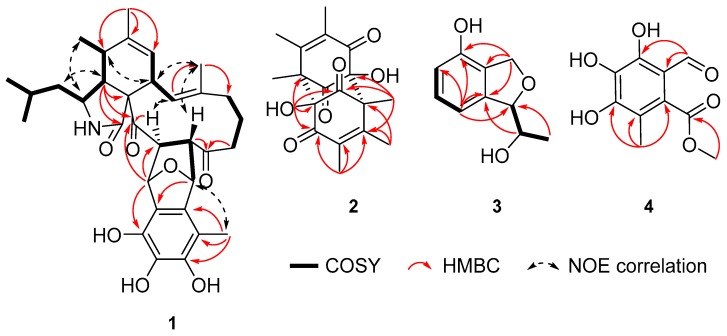
The key 2D NMR correlations of **1**–**4**.

Asperchalasine I (**1**), the molecular formula, C_33_H_41_O_7_N, was determined on the basis of the HRESIMS ion at *m*/*z* 562.2798 ([M − H]^−^ calcd. for C_33_H_40_O_7_N: 562.2799). As shown in Table 1, the ^1^H NMR data indicated characteristic signals of two double-bond protons (*δ*_H_ 5.93 and 5.36), nine methine protons (*δ*_H_ 5.52, 5.06, 4.46, 3.76, 3.21, 3.05, 2.88, 2.54 and 1.62), four methylene protons (*δ*_H_ 2.09, 1.90, *δ*_H_ 2.01, 1.58, *δ*_H_ 1.35 and *δ*_H_ 1.21) and six methyl groups (*δ*_H_ 2.05, 1.78, 1.26, 1.17, 0.96 and 0.94). Subsequently, the ^13^C NMR data showed the presence of 33 carbon signals, according to the DEPT and HSQC data, which were identified as three carbonyls (*δ*_C_ 211.6, 203.6 and 176.3), an aromatic ring (*δ*_C_ 141.3, 133.7, 132.6, 132.5, 123.7 and 111.9), two trisubstituted double bonds (*δ*_C_ 141.8, 137.4, 125.8 and 125.1), nine methines, four methylenes, six methyls and a quaternary carbon (*δ*_C_ 67.3). The ^1^H-^1^H COSY (Figure 2) correlations of H_3_-23/H-22/H_2_-10/H-3/H-4/H-5/H_3_-11, H-7/H-8/H-13, H_2_-15/H_2_-16/H_2_-17 and H-19/H-20, together with the HMBC correlations from H-4 to C-1, C-9 and C-21, from H_3_-12 to C-5, C-6 and C-7, from H-13 to C-25, from H_3_-25 to C-15, from H_2_-17 and H-19 to C-18, and from H-20 to C-21, suggested the presence of an cytochalasan moiety. Meanwhile, the epicoccine moiety was inferred by HMBC correlations from H-1′ to C-3′, from H-8′ to C-2′ and from H_3_-9′ to C-5′, C-6′ and C-7′. The key linking relation of the cytochalasan moiety and epicoccine moiety through C-19/C-8′ and C-20/C-1′ C-C bonds was suggested from the ^1^H-^1^H COSY cross-peak of H-19/H-8′ and the HMBC correlation from H-1′ to C-20 and C-21. Moreover, the structure of **1** was further confirmed by a detailed comparison of the NMR data of **1** and asperchalasine B [26], which suggested that they shared the same skeleton. The upfield shifted of H_2_-17 (*δ*_H_ 1.21) in **1** (*δ*_H_ 4.10 in asperchalasine B) and the disappearance of a methoxy group at C-4′ (*δ*_H_ 3.70, *δ*_C_ 61.0), suggesting the replacements of methine at C-17 and a methoxy group at C-4′ in asperchalasine B with a methylene and hydroxy group in **1**, respectively.

**Figure 3:**
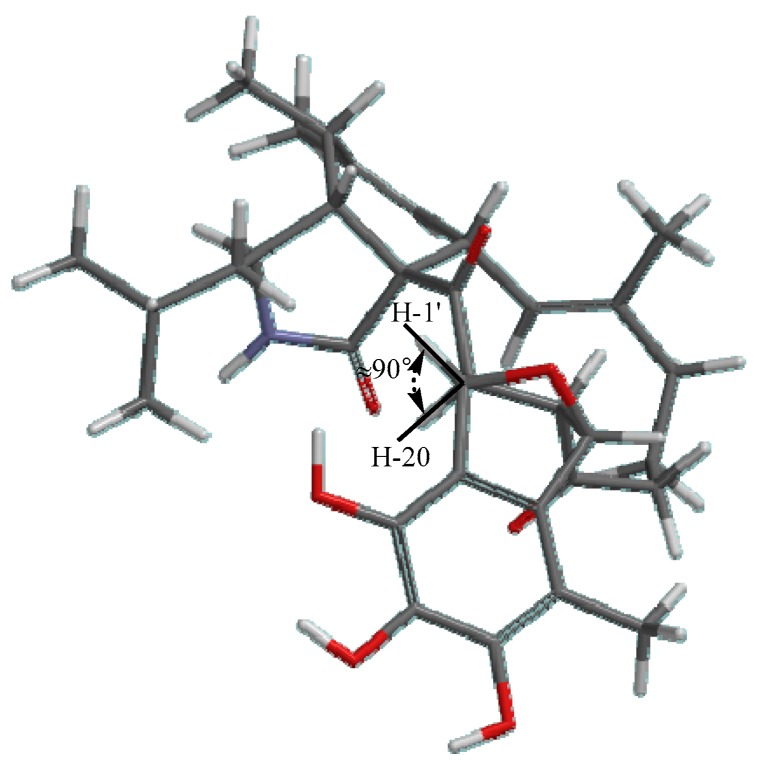
Molecular model of **1** (1′*β*, 8′ *β*-oxygen bridge).

Detailed analysis of NOESY data determined the relative configuration of chiral carbons in compound **1**. The NOESY correlations of H-4/H_2_-10, H-3/H_3_-11, H-5/H-8/H_3_-25, H-13/H-20 and H-19/H_3_-25 suggested that these protons were cofacial. Neither ^1^H-^1^H coupling nor a ^1^H-^1^H COSY correlation was observed between the protons of H-20 (*δ*_H_ 4.46, d, *J* = 5.8 Hz) and H-1′ (*δ*_H_ 5.06, s), which suggested that the dihedral angle of those protons was approximately 90° (Figure 3) [26]. The electron circular dichroism (ECD) spectrum (Figure S8) showed two positive Cotton effects (CE) at 228 nm and 306 nm, which were also consistent with those of asperchalasine B. Therefore, the absolute configuration of **1** was suggested to be 3*S*, 4*R*, 5*S*, 8*S*, 9*S*, 19*S*, 20*S*, 1′*S*, 8′*R*.

**Figure 4:**
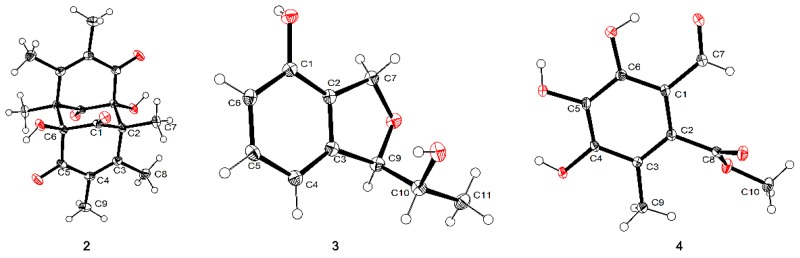
Single-crystal X-ray structures of **2**–**4**.

Dibefurin B (**2**), a colorless monocrystal, was assigned a molecular formula of C_18_H_20_O_6_ by the HRESIMS ion at *m*/*z* 331.1187 ([M − H]^−^ calcd. for C_18_H_19_O_6_: 331.1187). For the monomer, the ^1^H, ^13^C NMR (Table 2) and HSQC data of **2** revealed diagnostic signals for nine carbons, including two carbonyls (*δ*_C_ 200.6 and 192.6), two disubstituted olefin carbons (*δ*_C_ 157.6 and 131.4), an oxygen-bearing carbon [*δ*_H_ 6.67 (OH-6), *δ*_C_ 91.7 (C-6)], three methyls (*δ*_C_ 19.1, 12.2 and 12.1) and a quaternary carbon (*δ*_C_ 60.4). The HMBC correlations from H_3_-7 to C-1, C-2, C-3 and C-6′, from H_3_-8 to C-2, C-3 and C-4, from H_3_-9 to C-3, C-4 and C-5, revealed that Me-7, Me-8 and Me-9 were connected at C-2, C-3 and C-4, respectively. Similarly, the hydroxyl was attached to C-6, as evidenced by the HMBC correlations from OH-6 to C-1, C-2′, C-5 and C-6. In view of the HRESIMS data and X-ray (Figure 4), it could confirm that compound **2** was a symmetric dimer and the absolute configuration of **2** was assigned as 2*S*, 6*R*, 2′*R*, 6′*S*.

Compound **3** was purified as a colorless crystal whose molecular formula was deduced as C_10_H_12_O_3_ based on HRESIMS data (179.0716 [M − H]^−^, calcd. 179.0714). Analysis of the ^1^H NMR spectrum of **3** (Table 3) displayed three aromatic proton resonances (*δ*_H_ 7.13, 6.82 and 6.69), an oxygenated methylene (*δ*_Ha_ 5.12, *δ*_Hb_ 5.02), two oxygenated methines (*δ*_H_ 5.06 and 3.97), and a methyl (*δ*_H_ 1.20). The ^13^C spectrum revealed 10 signals, indicating an aromatic ring, a methylene, two methines and a methyl. In the ^1^H-^1^H COSY spectrum, the ortho-trisubstitution on the aromatic ring was confirmed by the cross-peaks of H-4/H-5/H-6. Moreover, the ^1^H-^1^H COSY spectrum showed correlations from H-10 to H-9 and H-11, and the chemical shift of C-10 (*δ*_C_ 70.6) showed the hydroxyl was attached to C-10. Subsequently, the HMBC correlations between H-6 and C-1/C-2 determined the linkage of 1-OH to C-1, and the correlations between H-7 and C-1/C-2/C-3, H-9 and C-2/C-3/C-10 established the presence of a phthalan ring. The same relative configuration of C-9 and C-10 was clearly deduced under the guidance of single-crystal X-ray (Figure 4). Hence, the absolute configuration of **3** was determined as 9*R*, 10*R*.

Compound **4** was deduced to have a molecular formula of C_10_H_10_O_6_ from its HRESIMS spectrum with a deprotonated molecular ion at *m*/*z* 225.0407. The ^1^H NMR (Table 3) in MeOH-*d*_4_ showed three singlets at *δ*_H_ 9.71, 3.90 and 2.08, according to the ^13^C NMR and HSQC data, which were attributed to an aldehyde group (*δ*_C_ 194.7), a methoxy group (*δ*_C_ 52.9) and a methyl (*δ*_C_ 12.4), respectively. In addition, resonances of a carbonyl and an aromatic ring were observed in the ^13^C NMR data. In the HMBC spectrum, the correlations of H-9 to C-2, C-3 and C-4 supported the connection of Me-9 to C-3, the correlations of H-7 to C-1 and C-6 indicated the linkage of aldehyde group and C-1. Meanwhile, the carbonyl (*δ*_C_ 169.9, C-8) had the HMBC correlation from H-10 (*δ*_H_ 3.90), further indicated the presence of methyl ester. With the assistance of single-crystal X-ray (Figure 4), the structure of compound **4** was clearly confirmed.

### 2.2. Biological Evaluation

Compounds **1**–**11** were tested for their inhibitory effects against α-glucosidase, and antioxidant activity. As seen in Table 4, the results indicated that compounds **1**, **8** and **9** showed significant inhibitory effects against α-glucosidase with IC_50_ values of 17.1, 26.7 and 15.7 μM, respectively, which were better than the positive controls acarbose (610.2 μM) and 1-deoxynojirimycin (71.5 μM). Beyond that, all of the compounds were tested for their antioxidant activity based on DPPH· (2, 2-diphenyl-1-picrylhydrazyl radical) scavenging. The results showed the antioxidant activity of **8** was 89% at the concentration of 100 μM and compound **8** possessed more potent capacity than positive control ascorbic acid in scavenging DPPH· with an EC_50_ value of 16.3 μM. Compounds **1**, **4** and **6** also exhibited weak DPPH· scavenging activity with respective EC_50_ values of 77.8, 85.8 and 59.1 μM.

## 3. Experimental Section

### 3.1. General Experimental Procedures

UV data were measured on a UV-Vis-NIR spectrophotometer (Perkin Elmer, Waltham, UK). IR spectrum data were recorded using a Bruker Vector spectrophotometer 22. Melting points were tested on a Fisher-Johns hot-stage apparatus which were uncorrected. Optical rotations were recorded using an MCP300 (Anton Paar, Shanghai, China). HRESIMS data were conducted on an Ion Mobility-Q-TOF High-Resolution LC-MS (Synapt G2-Si, Waters). The ECD experiment data were measured with J-810 spectropolarimeter (JASCO, Tokyo, Japan). The NMR spectra were recorded on Bruker Avance spectrometer (Bruker, Beijing, China) (Compounds **1** and **3**: 500 MHz for ^1^H and 125 MHz for ^13^C, respectively; compounds **2** and **4**: 400 MHz for ^1^H and 100 MHz for ^13^C). Column chromatography (CC) was carried out on silica gel (200–300 mesh, Marine Chemical Factory, Qingdao, China) and sephadex LH-20 (Amersham Pharmacia, Piscataway, NJ, USA).

### 3.2. Fungal Materials

The fungus used in this research was isolated from the fruit of the marine mangrove plant *Bruguiera* collected in 2014 in Hainan Dongzhai Harbor Mangrove Reserve by using the standard protocol. The strain was identified as *Mycosphaerella* sp. (compared to no. KX067865.1) upon the analysis of ITS sequence data of the rDNA gene. The ITS sequence data obtained from the fungal strain has been submitted to GenBank with accession no. MN194208. A voucher strain was deposited in our laboratory.

### 3.3. Fermentation, Extraction and Isolation

The fungus *Mycosphaerella* sp. SYSU-DZG01 was grown on solid cultured medium in 100 × 1000 mL Erlenmeyer flasks at room temperature for 30 days under static conditions, each containing 80 g rice and 120 mL 0.3% saline. After incubation, the former was extracted with methanol twice and concentrated to yield 10.9 g of crude extract under reduced pressure. The crude extract was subjected to LC-HRESIMS analysis (Figure S29). Then, the residue was eluted by using gradient elution with petroleum ether/EtOAc from 9:1 to 0:10 (*v*/*v*) on silica gel CC to get ten fractions (Fr.1–Fr.10). Fr.2 (630 mg) was further eluted by silica gel CC using CH_2_Cl_2_/MeOH (40:1) to obtain Fr.2.1–Fr.2.3. Fr.2.3 (301 mg) was purified by Sephadex LH-20 CC and eluted with MeOH to obtain compound **2** (3.5 mg), **6** (11.1 mg) and 1**0** (3.6 mg). Fr.4 (217 mg) was applied to silica gel CC by CH_2_Cl_2_/MeOH (20:1) to obtain Fr.4.1–Fr.4.7. Fr.4.1 (8.1 mg) was further purified by Sephadex LH-20 CC using MeOH to obtain compound **1** (2.3 mg), **3** (2.2 mg), **8** (20.8 mg) and **9** (2.7 mg). Fr.5 (817 mg) was eluted (by CH_2_Cl_2_/MeOH, 25:3) to obtain Fr.5.1–Fr.5.5. Fr.5.1 (13.3 mg), Fr.5.2 (27.7 mg) and Fr.5.4 (10.9 mg) was purified by Sephadex LH-20 CC using CH_2_Cl_2_/MeOH (1:1) to yield compound **4** (4.3 mg), **5** (2.0 mg), **7** (3.7 mg) and **11** (2.7 mg).

Asperchalasine I (**1**): White powder; [α${]}_{D}^{25}$ = +61.4 (*c* 0.1, MeOH); UV (MeOH) *λ*_max_ (log *ε*): 206 (4.53) nm; IR (KBr) *ν*_max_ (cm^−1^): 3369, 1691, 1440, 1384, 1201, 1120, 1053; HRESIMS *m*/*z* 562.2798 [M − H]^−^ (calcd. for C_33_H_40_O_7_N: 562.2799); ^1^H and ^13^C NMR data: see Table 1.

Dibefurin B(**2**): Colorless crystal; m.p. 67.8–69.2 °C; [α${]}_{D}^{25}$ = +0.3 (*c* 0.1, MeOH); UV (MeOH) *λ*_max_ (log *ε*): 237 (3.98) nm; IR (KBr) *ν*_max_ (cm^−1^): 3448, 1747, 1664, 1645, 1238, 1037; HRESIMS *m*/*z* 331.1187 [M − H]^−^ (calcd. for C_18_H_19_O_6_, 331.1187); ^1^H and ^13^C NMR data: see Table 2.

Compound **3**: Colorless crystal; m.p. 89.8–91.9 °C; [α${]}_{D}^{25}$ = −37.1 (*c* 0.1, MeOH); UV (MeOH) *λ*_max_ (log *ε*) 204 (4.39), 269 (3.24) nm; IR (KBr) *ν*_max_ (cm^−1^): 3261, 2887, 1601, 1471, 1297, 767, 706; HRESIMS *m*/*z* 179.0716 [M − H]^−^ (calcd. for C_10_H_12_O_3_, 179.0714; ^1^H and ^13^C NMR data: see Table 2.

Compound **4**: Colorless crystal; m.p. 95.9–97.8 °C; [α${]}_{D}^{25}$ = +3.4 (*c* 0.1, MeOH); UV (MeOH) *λ*_max_ (log *ε*) 241 (3.66), 305 (3.56) nm; IR (KBr) *ν*_max_ (cm^−1^): 3375, 2962, 1711, 1641, 1261, 1150, 933; HRESIMS *m*/*z* 225.0407 [M − H]^−^ (calcd. for C_10_H_10_O_6_, 225.0407; ^1^H and ^13^C NMR data: see Table 2.

### 3.4. X-Ray Crystallographic Data

Colorless crystals of compounds **2**–**4** were obtained from MeOH-CH_2_Cl_2_ at room temperature by slow volatilization, and examined on an Agilent Xcalibur Nova single crystal diffractometer with Cu Kα radiation. 

The crystallographic data for compound **2** has been deposited in the Cambridge Crystallographic Data Centre (CCDC number: 18022803)

Crystal data of **2**: C_18_H_20_O_6_, *Mr* = 332.34, triclinic, *a* = 6.9740(4) Å, *b* = 7.9800(4) Å, *c* = 14.3659(6) Å, *α* = 101.148(4)°, *β* = 99.209(4)°, *γ* = 98.479(5)°, *V* = 761.05(7)Å^3^; space group *P-1*, *Z* = 2, *Dc* = 1.450 g/cm^3^, *μ* = 0.908 mm^−1^ and *F*(000) = 352.0; Crystal dimensions: 0.40 × 0.30 × 0.02 mm^3^. Independent reflections: 4998 (*R*_int_ = 0.0269). The final *R*_1_ was 0.0522, *wR*_2_ = 0.1447 [*l* > 2*σ* (*I*)]. The goodness of fit on *F*^2^ was 1.048.

The crystallographic data for compound **3** has been deposited in the Cambridge Crystallographic Data Centre (CCDC number: 18121203)

Crystal data of **3**: C_10_H_12_O_3_, *Mr* = 180.07, monoclinic, *a* = 4.7697(1) Å, *b* = 11.1895(3) Å, *c* = 9.1541(3) Å, *α* = 90°, *β* = 93.829(3)°, *γ* = 90°, *V* = 487.47(2) Å^3^; space group *P21*, flack 0.14(18), *Z* = 2, *Dc* = 1.350 g/cm^3^, *μ* = 0.872 mm^−1^ and *F*(000) = 212.0; Crystal dimensions: 0.40 × 0.10 × 0.05 mm^3^. Independent reflections: 7438 (*R*_int_ = 0.0620). The final *R*_1_ was 0.0445, *wR*_2_ = 0.1258 [*l* > 2*σ* (*I*)]. The goodness of fit on *F*^2^ was 1.046.

The crystallographic data for compound **4** has been deposited in the Cambridge Crystallographic Data Centre (CCDC number: 18120705)

Crystal data of **4**: C_10_H_10_O_6_, *Mr* = 226.04, orthorhombic, *a* = 15.9208(7) Å, *b* = 6.6849(3) Å, *c* = 18.5162(7) Å, *α* = 90°, *β* = 90°, *γ* = 90°, *V* = 1970.66(14) Å^3^; space group *Pbca*, *Z* = 8, *Dc* = 1.525 g/cm^3^, *μ* = 1.108 mm^−1^ and *F*(000) = 944.0; Crystal dimensions: 0.25 × 0.03 × 0.03 mm^3^. Independent reflections: 3833 (*R*_int_ = 0.0496). The final *R*_1_ was 0.0485, *wR*_2_ = 0.1328 [*l* > 2*σ* (*I*)]. The goodness of fit on *F*^2^ was 1.050.

### 3.5. Biological Assays

#### 3.5.1. Inhibitory Activity of α-Glucosidase

The α-glucosidase inhibitory activity was assayed according to the reported method [29]. The inhibitory activity of α-glucosidase was tested in the 96-well plated with 100 mm PBS (KH_2_PO_4_-K_2_HPO_4_, pH 7.0) buffer solution each. Compounds **1**–**11**, acarbose and 1-deoxynojirimycin (positive control) were dissolved in DMSO, the substrate (*p*-nitrophenyl glycoside, 5 mM) were dissolved in PBS buffer solution and enzyme solutions (2.0 units/mL) were prepared. The assay was conducted in a 100 μL reaction system containing 20 μL enzyme stock solution, 69 μL PBS buffers and 1 μL of DMSO or testing materials. After 10 min incubation at 37 °C, 10 μL of the substrate was added and incubated for 20 min at 37 °C. The Absorbance which measured by a BIO-RAD (iMark) microplate reader at 405 nm was used to calculate the inhibitory activity according to the equation:

η (%) = [(B − S)/B] × 100%(1)

η (%) is the percentage of inhibition; B is the assay medium with DMSO; S is the assay medium with compound. The results of IC_50_ values were calculated by the nonlinear regression analysis. Acarbose and 1-deoxynojirimycin were used as positive controls.

#### 3.5.2. Antioxidant Activity

The DPPH· scavenging was assayed according to the reported method [30]. The DPPH radical scavenging test was performed in 96-well microplates. Testing materials (compounds **1**–**11**) were added to 150 μL (0.16 mmol/L) DPPH solution in MeOH at a range of 50 μL solutions of different concentrations (2, 25, 50 and 100 *μ*M). After 30 min, absorbance at 517 nm was measured and the percentage of activity was calculated. Ascorbic acid was used as a positive control.

## 4. Conclusions

In summary, four new metabolites, including one new asperchalasine I (**1**), dibefurin B (**2**), two epicoccine derivatives (**3**,**4**) and seven known compounds were isolated from the fungus *Mycosphaerella* sp. SYSU-DZG01. The structures of **1**–**11** were established by spectroscopic data and the absolute configuration of compounds **1**–**3** was determined in this research. Compound **1** possesses a unique T-shaped skeleton. All of the compounds were tested for their biological activities. Compounds **1**, **8** and **9** exhibited inhibitory effects against α-glucosidase with IC_50_ values of 17.1, 26.7 and 15.7 μM, respectively while compounds **1**, **4**, **6** and **8** showed antioxidant activity by scavenging DPPH· with EC_50_ values of 77.8, 85.8, 59.1 and 16.3 μM. These results suggested that the asperchalasine I may be a potential candidate for α-glucosidase inhibitor.

## Figures and Tables

**Table 1:** ^1^H (500 MHz) and ^13^C (125 MHz) NMR data for **1** in CDCl_3_.

Position	1	
	*δ*_H_, mult (*J* in Hz)	*δ*_C_, Type
1		176.3, C
2		
3	3.21, m	51.9, CH
4	3.05, dd (3.9, 5.0)	49.8, CH
5	2.54, m,	35.2, CH
6		141.8, C
7	5.36, s	125.1, CH
8	2.88, d (11.1)	43.3, CH
9		67.3, C
10	1.35, t	48.5, CH_2_
11	1.26, d (7.3)	13.6, CH_3_
12	1.78, s	20.1, CH_3_
13	5.93, d (11.0)	125.8, CH
14		137.4, C
15	2.09, s	40.7, CH_2_
	1.90, d (4.3)	
16	2.01, m	22.5, CH_2_
	1.58, m	
17	1.21, m	35.1, CH_2_
18		211.6, C
19	3.76, t (5.3)	56.9, CH
20	4.46, d (5.8)	57.3, CH
21		203.6, C
22	1.62, m	25.3, CH
23	0.94, dd (1.2, 6.5)	23.6, CH_3_
24	0.96, dd (1.2, 6.5)	21.3, CH_3_
25	1.17, s	14.2, CH_3_
1′	5.06, s	80.7, CH
2′		123.7, C
3′		132.5, C
4′		133.7, C
5′		141.3, C
6′		111.9, C
7′		132.6, C
8′	5.52, d (4.9)	81.3, CH
9′	2.05, s	11.9, CH_3_

**Table 2:** ^1^H (400 MHz) and ^13^C (100 MHz) NMR data for **2** in DMSO-*d*_6_.

Position	2	
	*δ*_H_, mult (*J* in Hz)	*δ*_C__,_ Type
1 (1′)		200.6, C
2 (2′)		60.4, C
3 (3′)		157.6, C
4 (4′)		131.4, C
5 (5′)		192.6, C
6 (6′)		91.7, C
7 (7′)	1.21, s	12.1, CH_3_
8 (8′)	2.02, s	19.1, CH_3_
9 (9′)	1.73, s	12.2, CH_3_

**Table 3:** ^1^H and ^13^C NMR data for **3** and **4** in MeOH-*d*_4_.

Position	3 ^a^		4 ^b^	
	*δ*_H_, mult (*J* in Hz)	*δ*_C,_ Type	*δ*_H_, mult (*J* in Hz)	*δ*_C_, Type
1		152.8, C		112.2, C
2		127.3, C		117.4, C
3		142.1, C		129.9, C
4	6.82, d (7.4)	114.3, CH		152.5, C
5	7.13, t (7.7)	129.9, CH		134.1, C
6	6.69, d (7.9)	115.0, CH		151.2, C
7	5.12, dd (2.8, 11.8)	72.4, CH_2_	9.71, s	194.7, CH
	5.02, d (11.9)			
8				169.9, C
9	5.06, d (3.3)	89.4, CH	2.08, s	12.4, CH_3_
10	3.97, qd (3.9, 6.4)	70.6, CH	3.90, s	52.9, CH_3_
11	1.20, d (6.4)	18.6, CH_3_		

^a 1^H and ^13^C NMR recorded at 500 MHz and 125 MHz; ^b 1^H and ^13^C NMR recorded at 400 MHz and 100 MHz.

**Table 4:** The α-glucosidase inhibitory and antioxidant activities of compounds **1**–**11**.

Compounds	α-Glucosidase Inhibitory	Antioxidant	
	IC_50_ (μM)	% Inhibition (100 μM)	EC_50_ (μM)
1	17.1	56	77.8
2	>50	<50	-
3	>50	<50	-
4	>50	57	85.8
5	>50	<50	-
6	>50	65	59.1
7	>50	<50	-
8	26.7	89	16.3
9	15.7	<50	-
10	-	-	-
11	>50	<50	-
Acarbose ^a^	610.2		
1-Deoxynojirimycin ^a^	71.5		
Ascorbic acid ^a^		92	22.4

- means no test; ^a^ positive control.

## Supplementary Materials

The following are available online at https://www.mdpi.com/1660-3397/17/8/483/s1, Figure S1: ^1^H NMR spectrum of compound **1** (500 MHz, CDCl_3_), Figure S2: ^13^C NMR spectrum of compound **1** (125 MHz, CDCl_3_), Figure S3: DEPT 135, DEPT 90 and ^13^C NMR spectrum of compound **1** (125 MHz, CDCl_3_), Figure S4: ^1^H-^1^H COSY spectrum of compound **1** (CDCl_3_), Figure S5: HSQC spectrum of compound **1** (CDCl_3_), Figure S6: HMBC spectrum of compound **1** (CDCl_3_), Figure S7: NOESY spectrum of compound **1** (CDCl_3_), Figure S8: HRESIMS spectrum of compound **1**, Figure S9: Experiment ECD spectrum of **1**, Figure S10: ^1^H NMR spectrum of compound **2** (400 MHz, DMSO-*d*_6_), Figure S11: ^13^C NMR spectrum of compound **2** (100 MHz, DMSO-*d*_6_), Figure S12: ^1^H-^1^H COSY spectrum of compound **2** (DMSO-*d*_6_), Figure S13: HSQC spectrum of compound **2** (DMSO-*d*_6_), Figure S14: HMBC spectrum of compound **2** (DMSO-*d*_6_), Figure S15: HRESIMS spectrum of compound **2**, Figure S16: ^1^H NMR spectrum of compound **3** (500 MHz, MeOH-*d*_4_), Figure S17: ^13^C NMR spectrum of compound **3** (125 MHz, MeOH-*d*_4_), Figure S18: ^1^H-^1^H COSY spectrum of compound **3** (MeOH-*d*_4_), Figure S19: HSQC spectrum of compound **3** (MeOH-*d*_4_), Figure S20: HMBC spectrum of compound **3** (MeOH-*d*_4_), Figure S21: NOESY spectrum of compound **3** (MeOH-*d*_4_), Figure S22: HRESIMS spectrum of compound **3**, Figure S23: ^1^H NMR spectrum of compound **4** (400 MHz, MeOH-*d*_4_), Figure S24: ^13^C NMR spectrum of compound **4** (100 MHz, MeOH-*d*_4_), Figure S25: ^1^H-^1^H COSY spectrum of compound **4** (MeOH-*d*_4_), Figure S26: HSQC spectrum of compound **4** (MeOH-*d*_4_), Figure S27: HMBC spectrum of compound **4** (MeOH-*d*_4_), Figure S28: HRESIMS spectrum of compound **4**. Figure S29: The LC-HRESIMS analysis profile of crude extract.
