# Supplementary material for: Secondary Metabolites with α-Glucosidase Inhibitory Activity from the Mangrove Fungus Mycosphaerella sp. SYSU-DZG01
Source: Mar Drugs. 2019 Aug 20;17(8):483. doi: 10.3390/md17080483 (PMC6723402; doi:10.3390/md17080483)
Supplement: Supplementary file 1 [file marinedrugs-17-00483-s001.pdf]

Secondary Metabolites with α-Glucosidase Inhibitory Activity From the Mangrove Fungus *Mycosphaerella* sp. SYSU-DZG01

Pei Qiu [1], Zhaoming Liu[1,2], Yan Chen[1], Runlin Cai[1], Guangying Chen[3,]*and Zhigang She [1,4]*

Affiliation

1 School of Chemistry, Sun Yat-sen University; Guangzhou 510275, China
2 State Key Laboratory of Applied Microbiology Southern China, Guangdong Institute of Microbiology, Guangdong Academy of Sciences, Guangzhou 510070, China
3 Key Laboratory of Tropical Medicinal Plant Chemistry of Ministry of Education, Hainan Normal University, Haikou 571158, China
4 South China Sea Bio-Resource Exploitation and Utilization Collaborative Innovation Center, Guangzhou 510006, China

* Correspondence:
E-mail address： chgying123@163.com (G-Y.C.); cesshzhg@mail.sysu.edu.cn (Z.S.)

Supporting Information Contents:

Fig.S1 ^{1}H NMR spectrum of compound **1** (500 MHz, CDCl3)..3
Fig.S2 ^{13}C NMR spectrum of compound **1** (125 MHz, CDCl3)..3
Fig.S3 DEPT 135, DEPT 90 and ^{13}C NMR spectrum of compound **1** (125 MHz, $CDCl_3$).....4
Fig.S4 ^{1}H-^{1}H COSY spectrum of compound **1** ($CDCl_3$)..4
Fig.S5 HSQC spectrum of compound **1** (CDCl3)..5
Fig.S6 HMBC spectrum of compound **1** ($CDCl_3$)..5
Fig.S7 NOESY spectrum of compound **1** ($CDCl_3$)..6
Fig.S8 HRESIMS spectrum of compound **1**..6
Fig.S9 Experiment ECD spectrum of compound **1**..7
Fig.S10 ^{1}H NMR spectrum of compound **2** (400 MHz, DMSO-d_6)..7
Fig.S11 ^{13}C NMR spectrum of compound **2** (100 MHz, DMSO-d_6)..8
Fig.S12 ^{1}H-^{1}H COSY spectrum of compound **2** (DMSO-d_6)..8
Fig.S13 HSQC spectrum of compound **2** (DMSO-d_6)..9
Fig.S14 HMBC spectrum of compound **2** (DMSO-d_6)..9
Fig.S15 HRESIMS spectrum of compound **2**..10
Fig.S16 ^{1}H NMR spectrum of compound **3** (500 MHz, MeOH-d_4)..10
Fig.S17 ^{13}C NMR spectrum of compound **3** (125 MHz, MeOH-d_4)..11
Fig.S18 ^{1}H-^{1}H COSY spectrum of compound **3** (MeOH-d_4)..11
Fig.S19 HSQC spectrum of compound **3** (MeOH-d_4)..12
Fig.S20 HMBC spectrum of compound **3** (MeOH-d_4)..12
Fig.S21 NOESY spectrum of compound **3** (MeOH-d_4)..13
Fig.S22 HRESIMS spectrum of compound **3**..13
Fig.S23 ^{1}H NMR spectrum of compound **4** (400 MHz, MeOH-d_4)..14
Fig.S24 ^{13}C NMR spectrum of compound **4** (100 MHz, MeOH-d_4)..14
Fig.S25 ^{1}H-^{1}H COSY spectrum of compound **4** (MeOH-d_4)..15
Fig.S26 HSQC spectrum of compound **4** (MeOH-d_4)..15
Fig.S27 HMBC spectrum of compound **4** (MeOH-d_4)..16
Fig.S28 HRESIMS spectrum of compound **4**..16
Fig.S29 The LC-HRESIMS analysis profile of crude extract...17

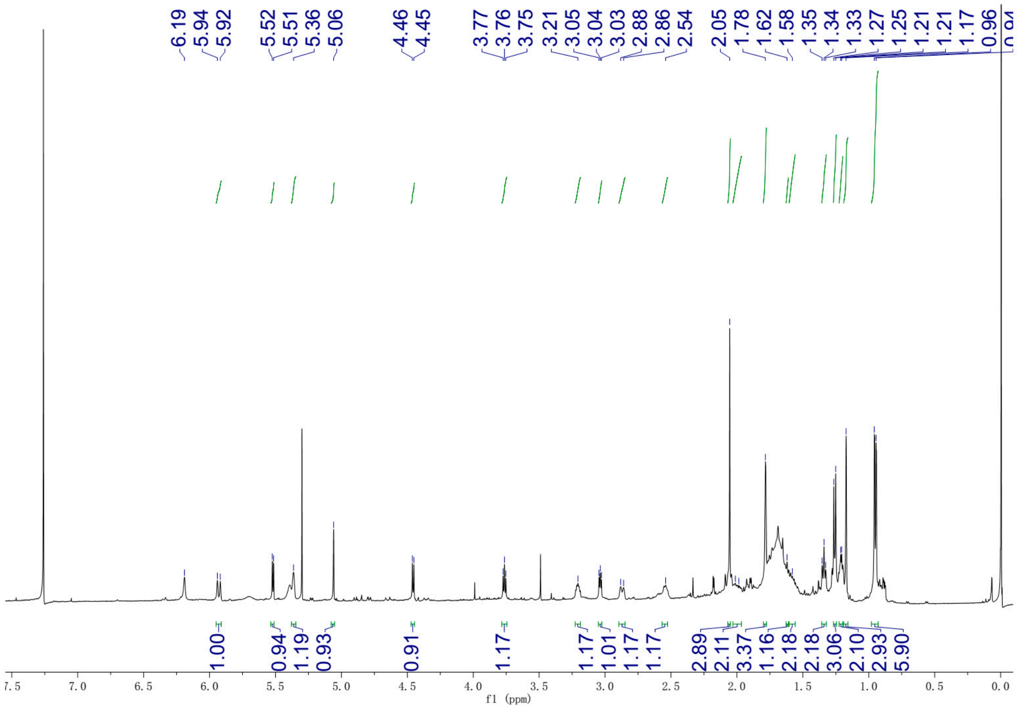

Fig.S1 ^{1}H NMR spectrum of compound **1** (500 MHz, CDCl3).

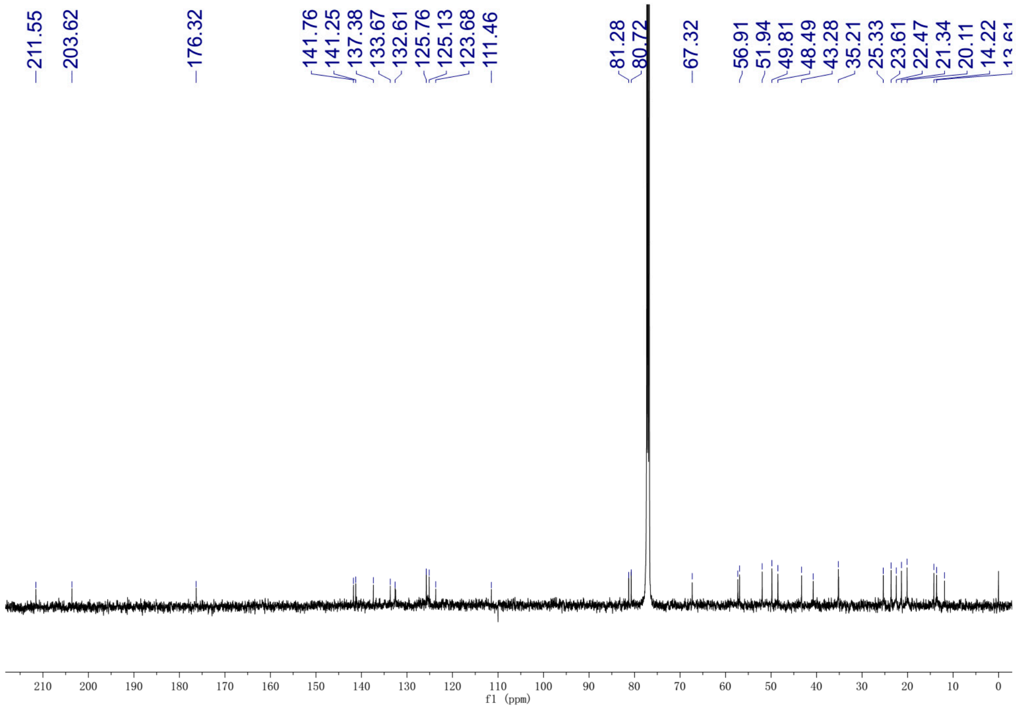

Fig.S2 ^{13}C NMR spectrum of compound **1** (125 MHz, CDCl3).

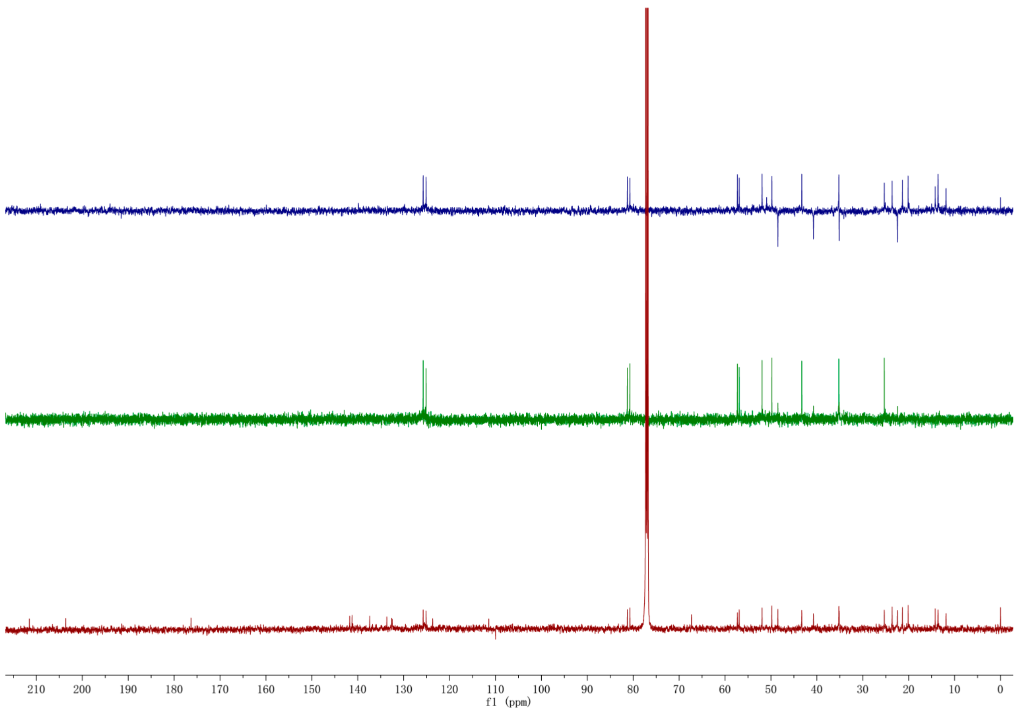

Fig.S3 DEPT 135, DEPT 90 and ^{13}C NMR spectrum of compound **1** (125 MHz, $CDCl_3$).

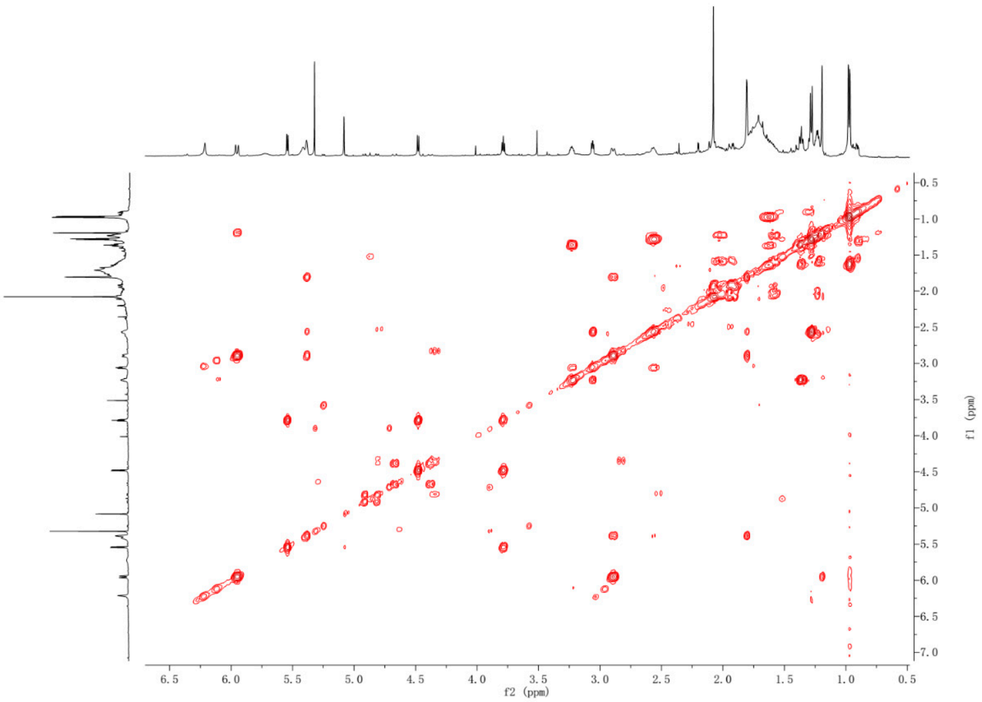

Fig.S4 ^{1}H-^{1}H COSY spectrum of compound **1** ($CDCl_3$).

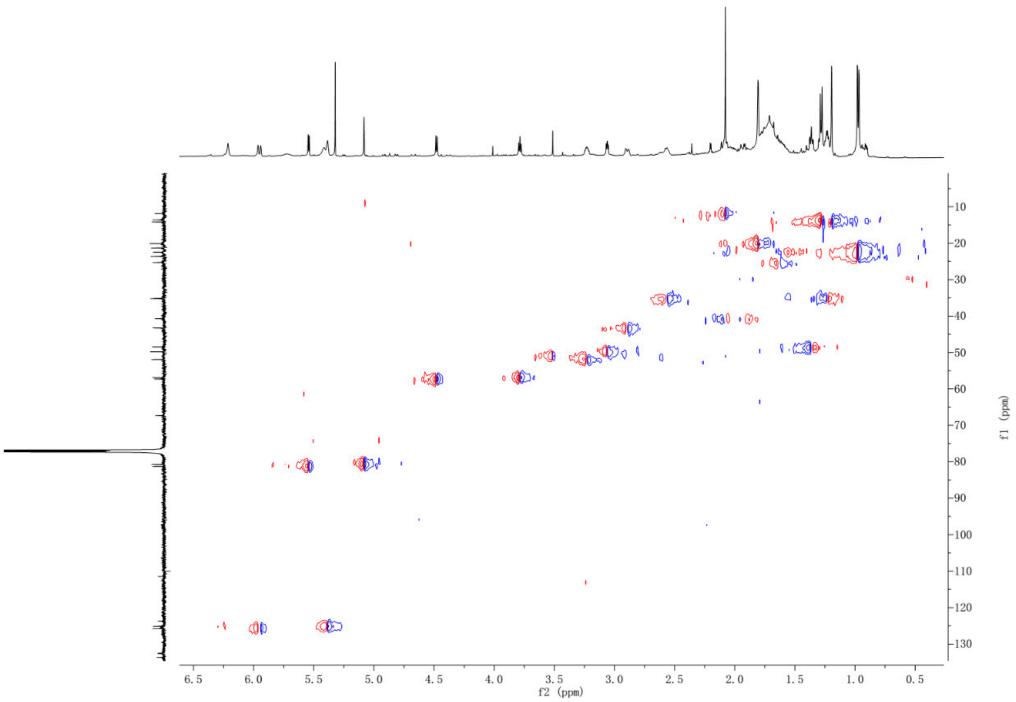

Fig.S5 HSQC spectrum of compound **1** (CDCl3).

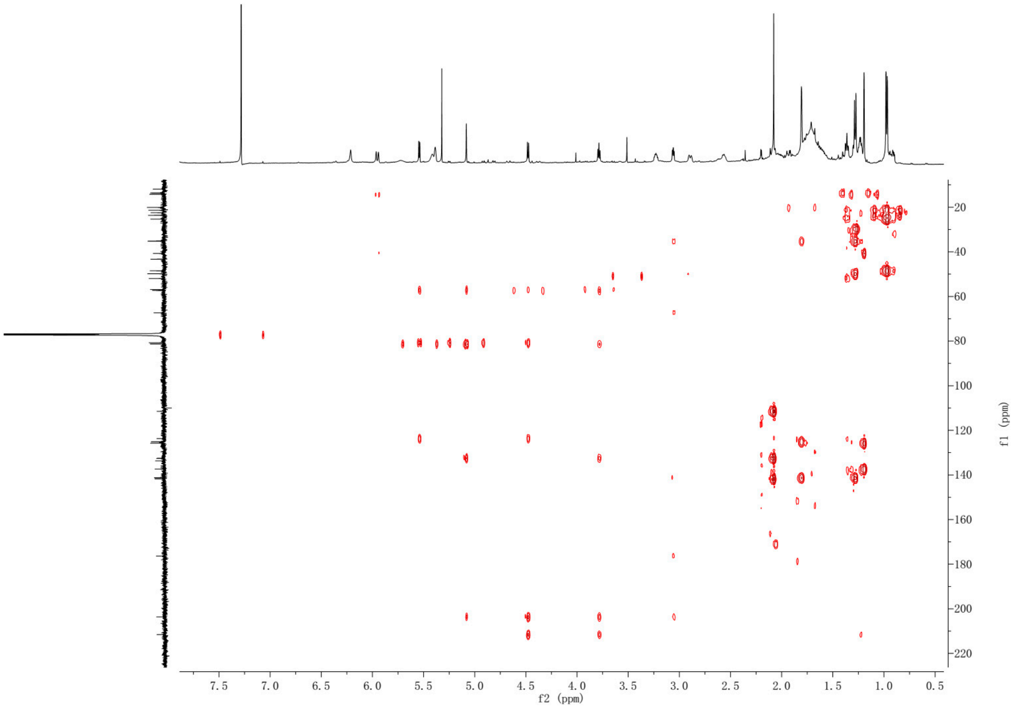

Fig.S6 HMBC spectrum of compound **1** ($CDCl_3$).

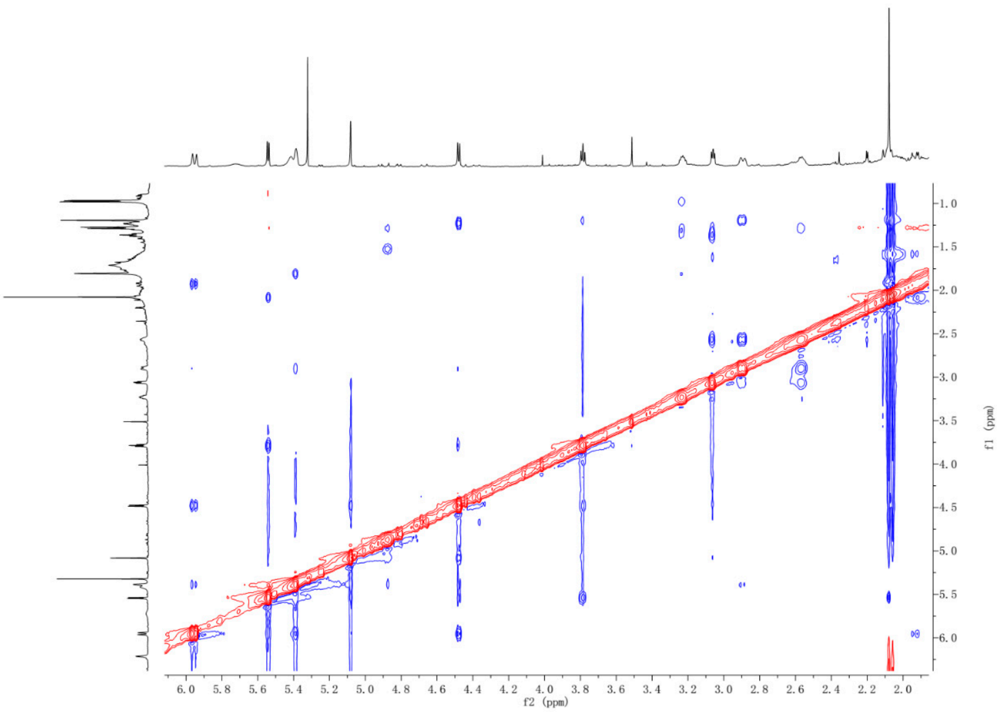

Fig.S7 NOESY spectrum of compound **1** ($CDCl_3$).

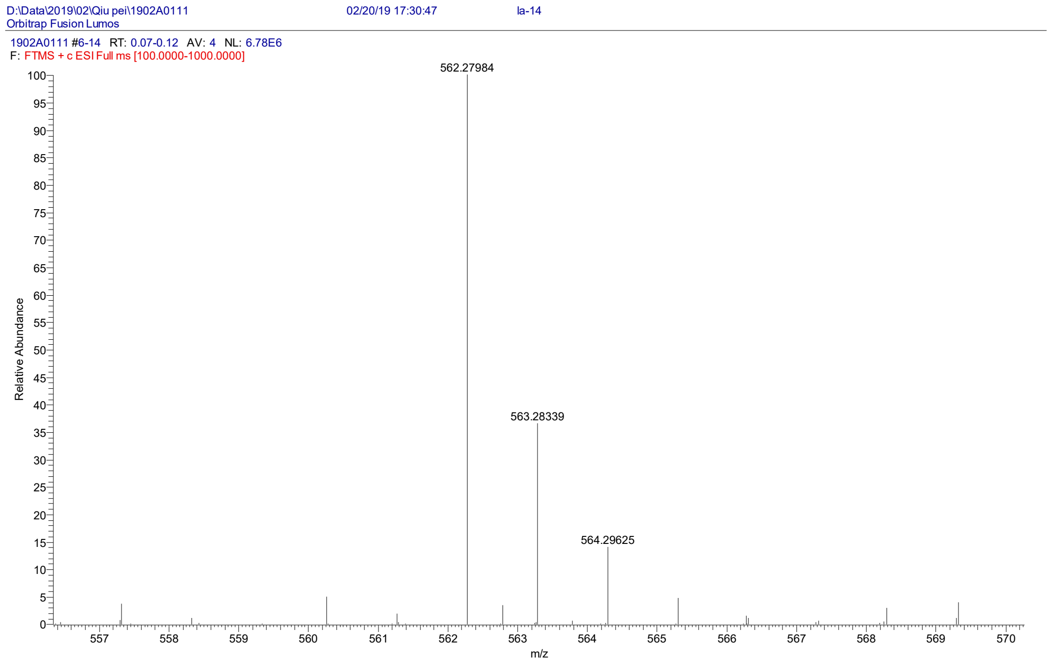

Fig.S8 HRESIMS spectrum of compound **1**.

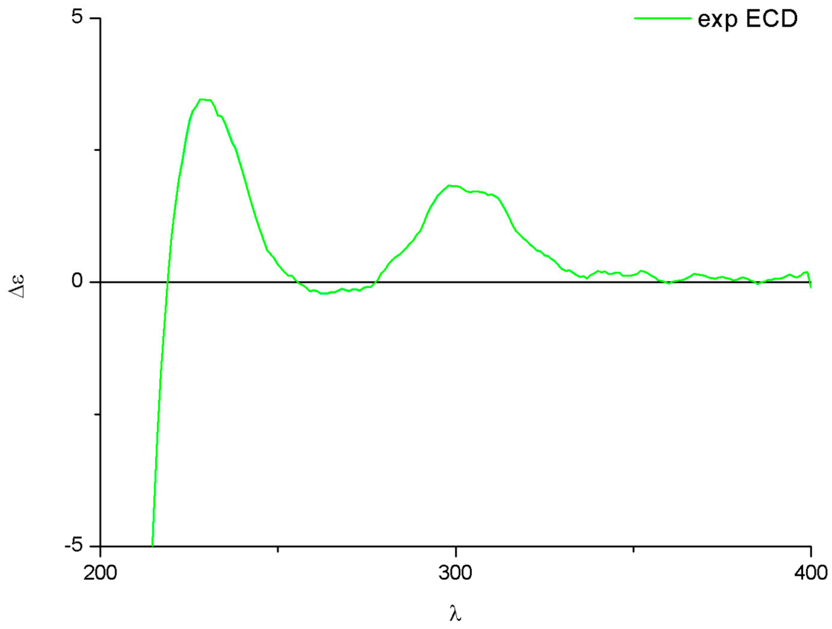

Fig.S9 Experiment ECD spectrum of compound **1**.

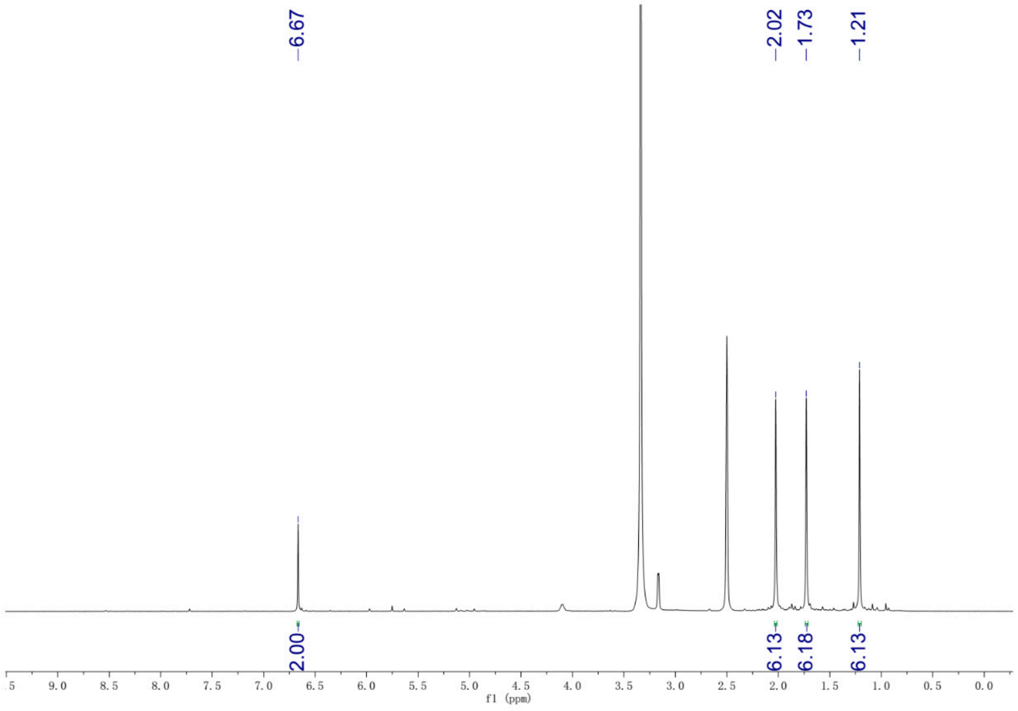

Fig.S10 ^{1}H NMR spectrum of compound **2** (400 MHz, DMSO-d_6).

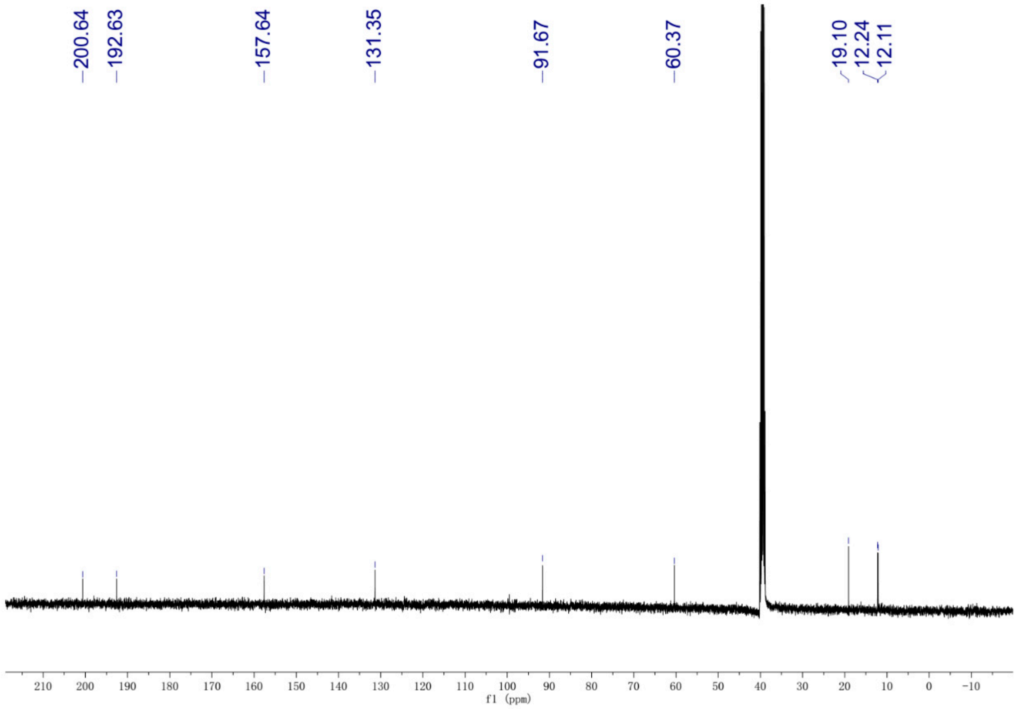

Fig.S11 ^{13}C NMR spectrum of compound **2** (100 MHz, DMSO-d_6).

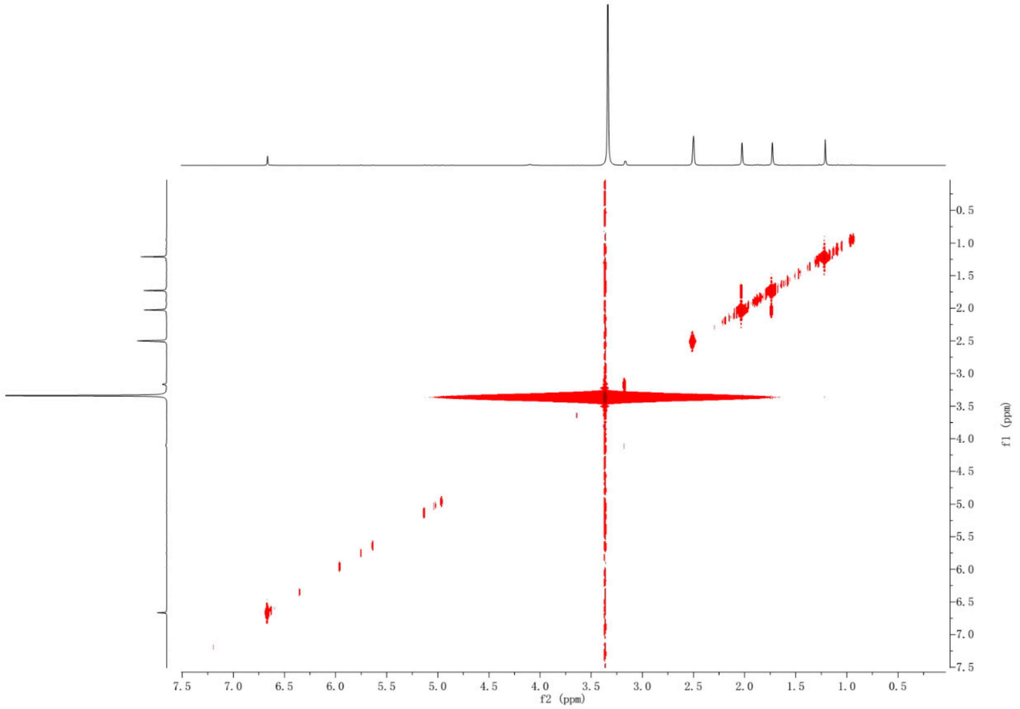

Fig.S12 ^1H-^1H COSY spectrum of compound **2** (DMSO-d_6).

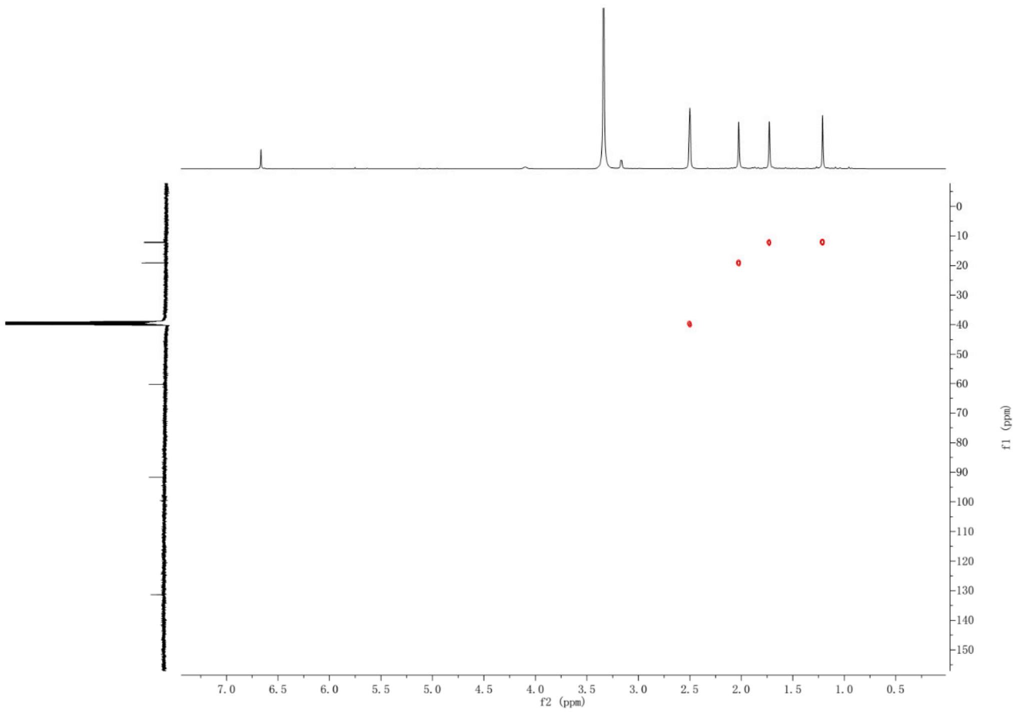

Fig.S13 HSQC spectrum of compound **2** (DMSO-*d*₆).

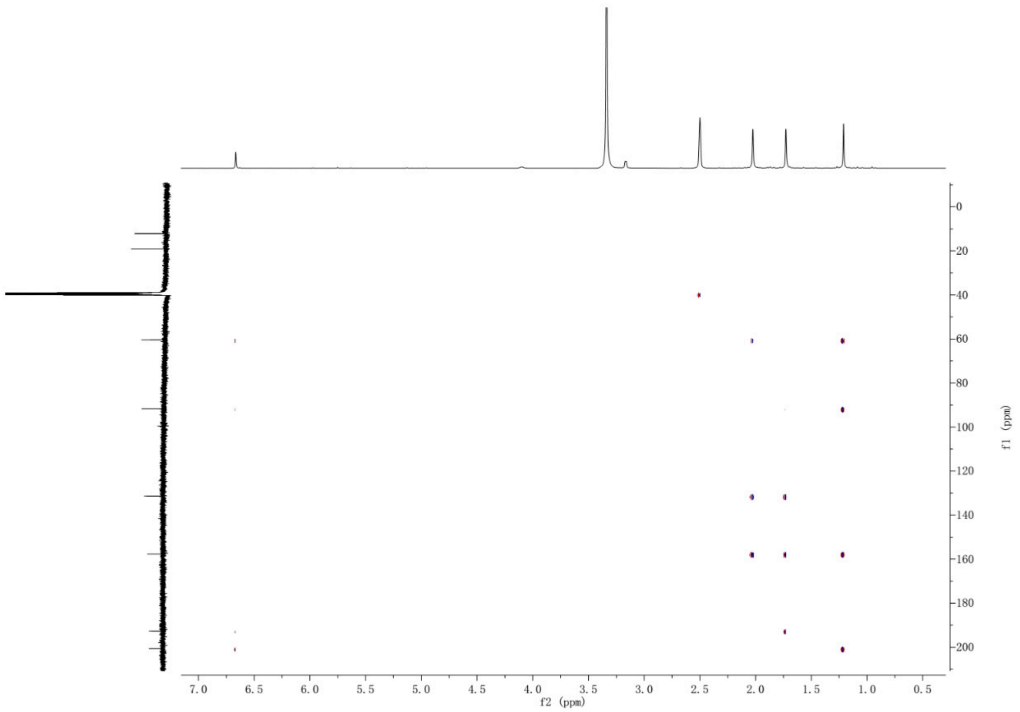

Fig.S14 HMBC spectrum of compound **2** (DMSO-*d*₆).

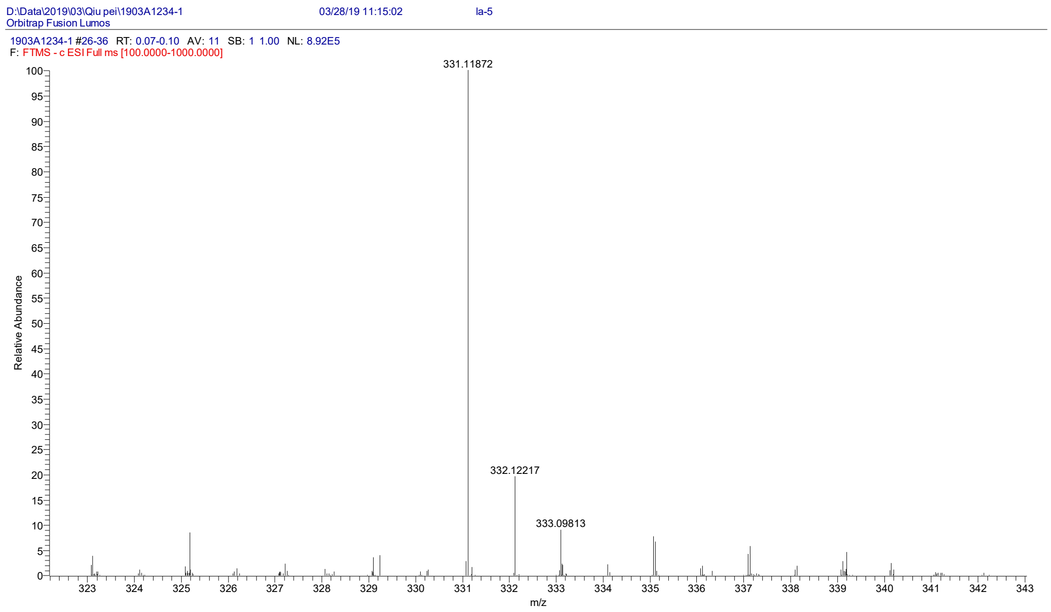

Fig.S15 HRESIMS spectrum of compound **2**.

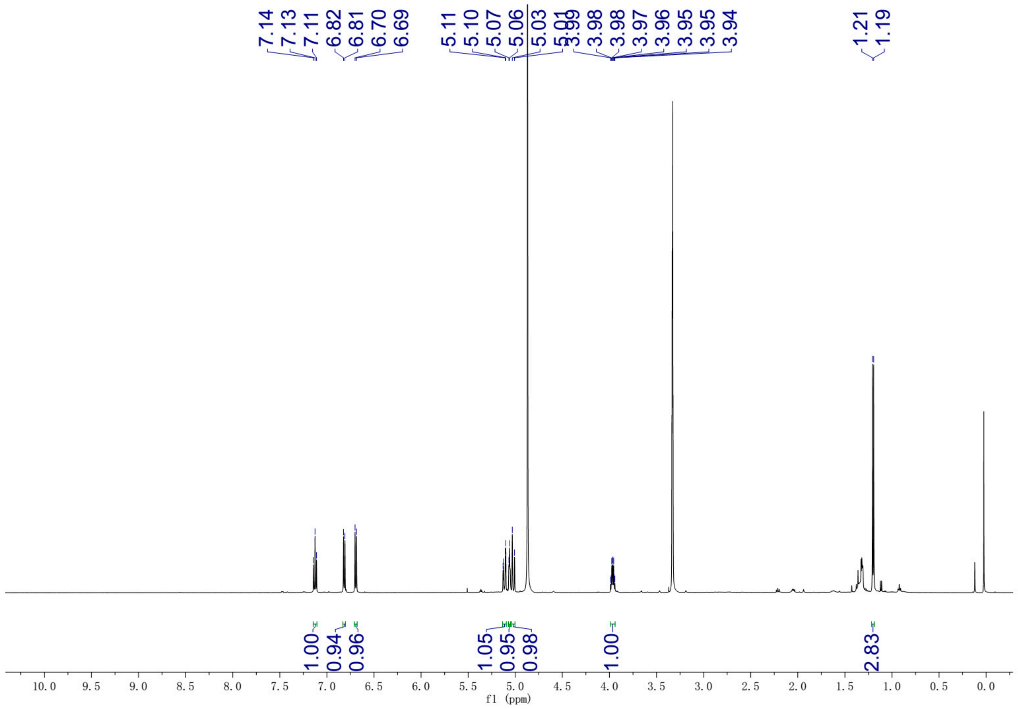

Fig.S16 ^{1}H NMR spectrum of compound **3** (500 MHz, MeOH-d_4).

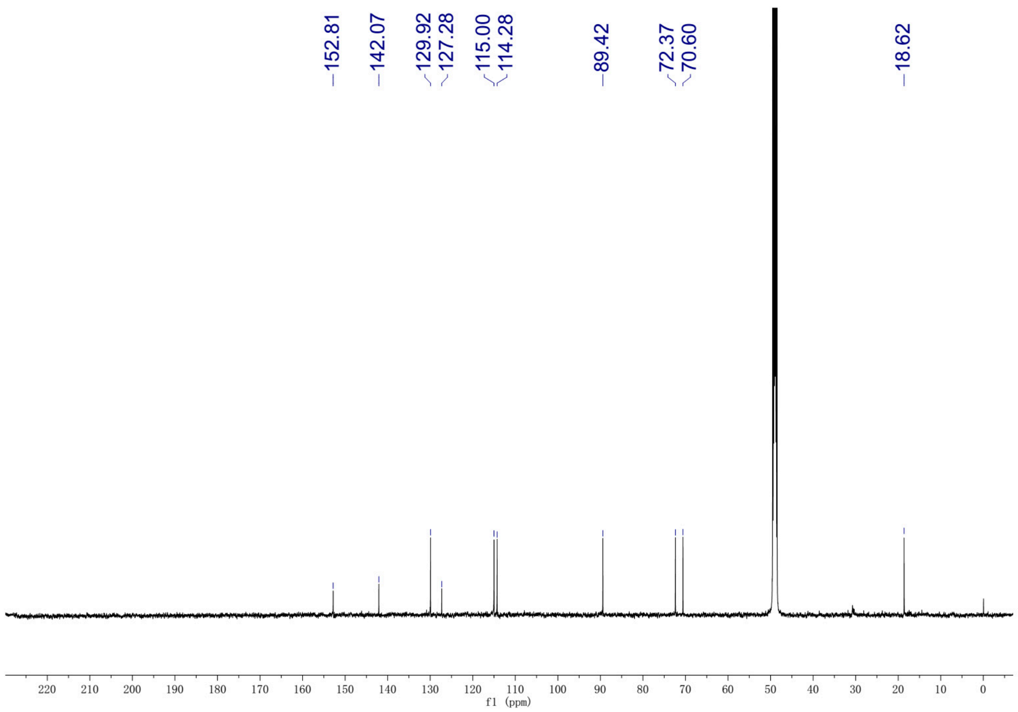

Fig.S17 ^{13}C NMR spectrum of compound **3** (125 MHz, MeOH-*d*$_4$).

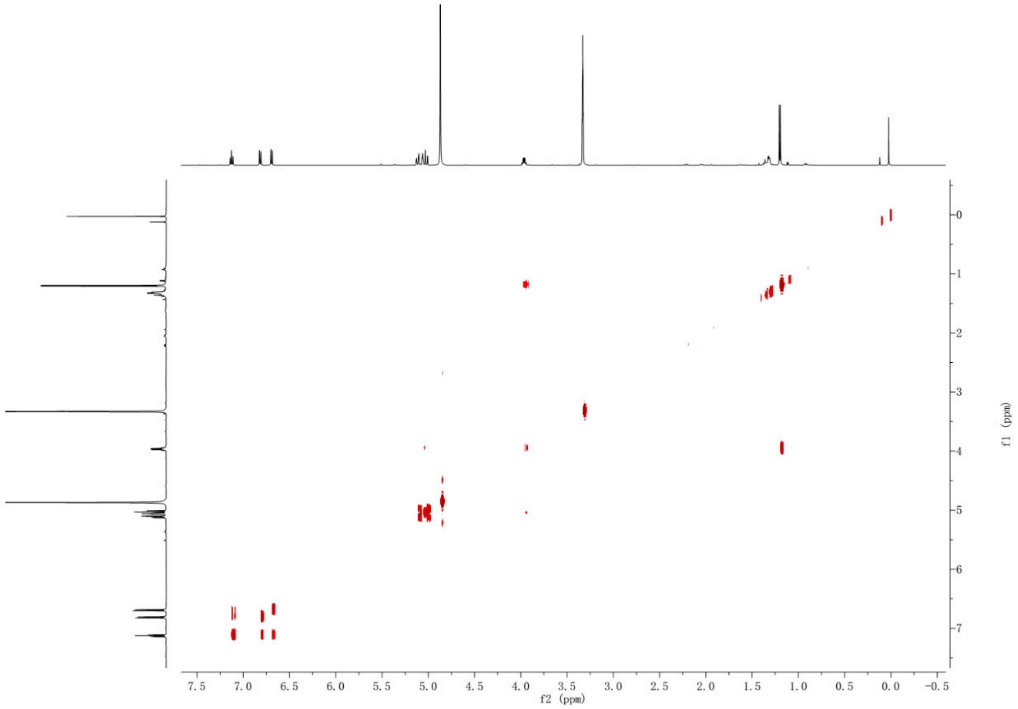

Fig.S18 ^{1}H-^{1}H COSY spectrum of compound **3** (MeOH-*d*$_4$).

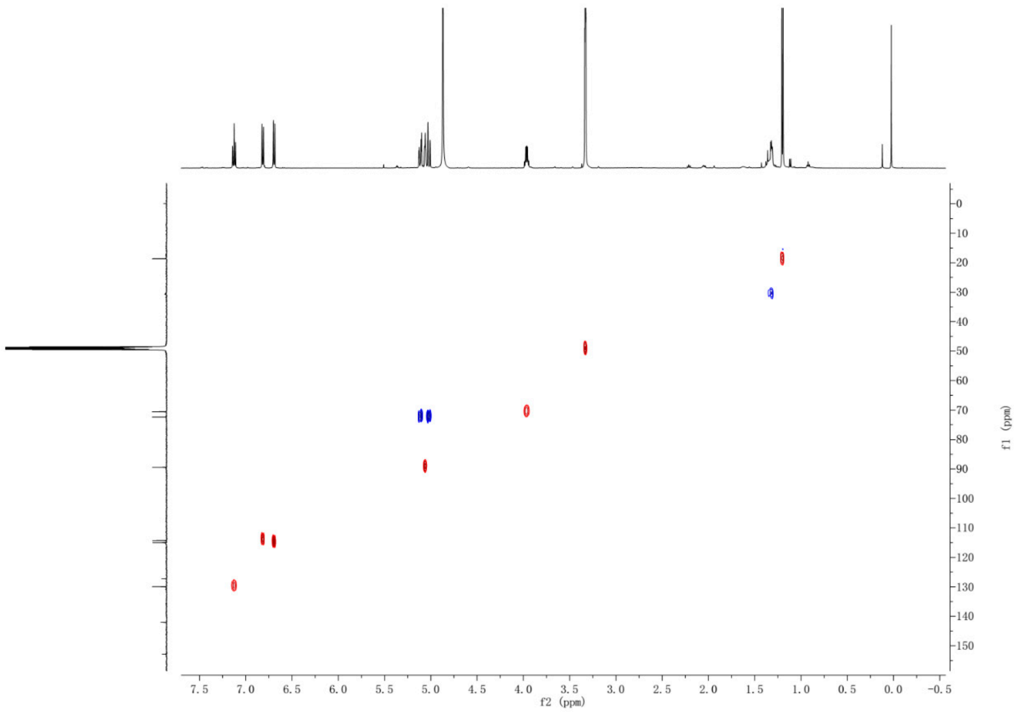

Fig.S19 HSQC spectrum of compound **3** (MeOH-d_4).

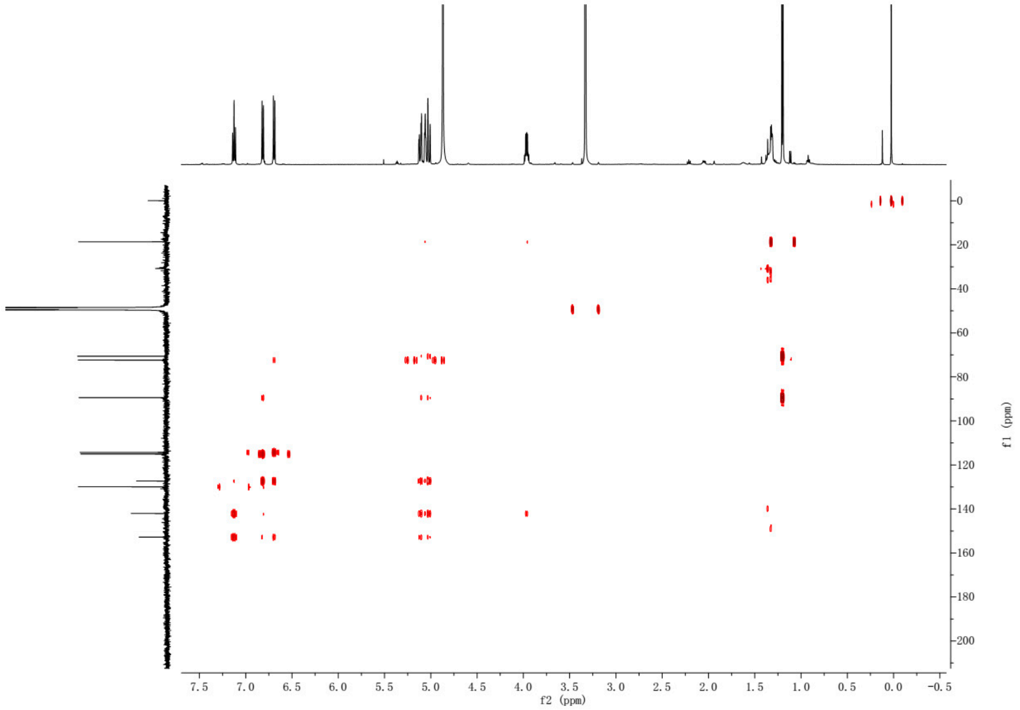

Fig.S20 HMBC spectrum of compound **3** (MeOH-d_4).

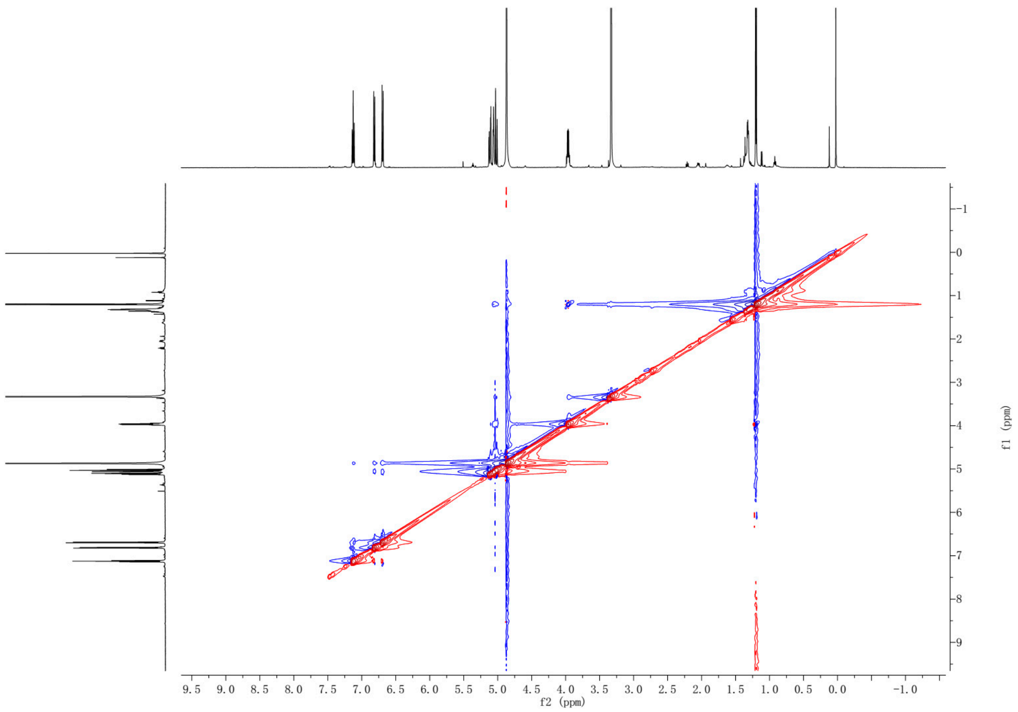

Fig.S21 NOESY spectrum of compound **3** (MeOH-d_4).

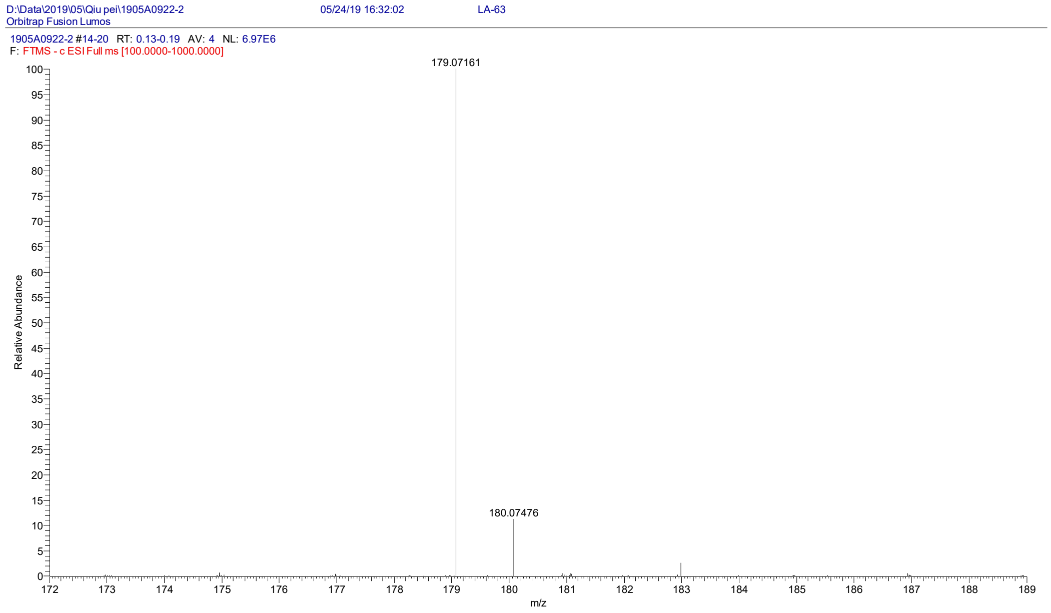

Fig.S22 HRESIMS spectrum of compound **3**.

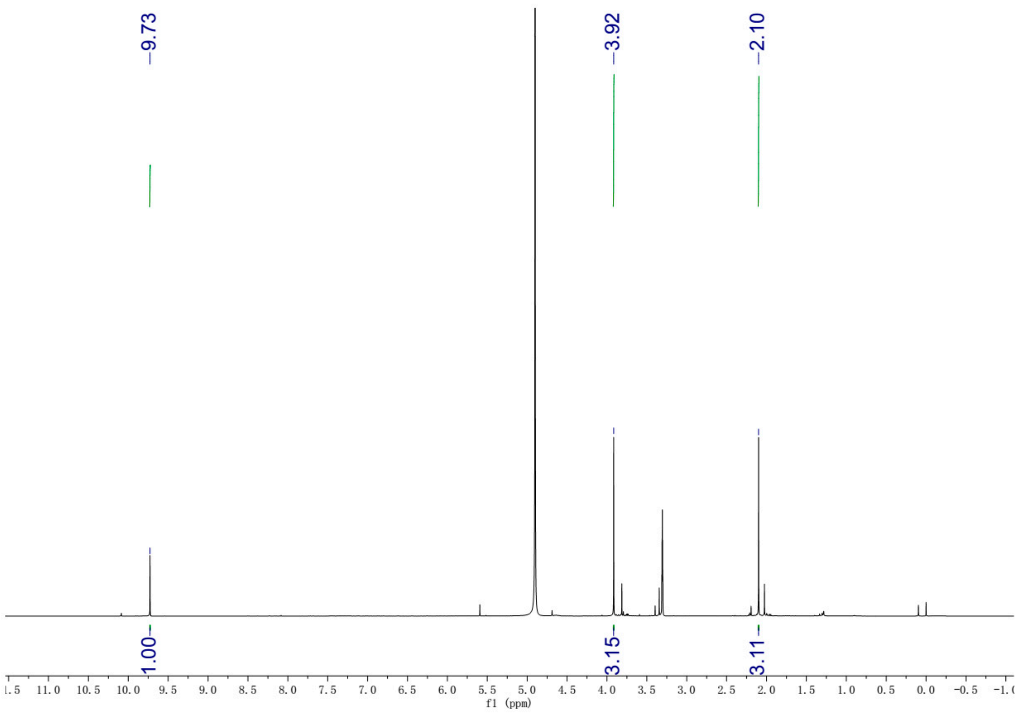

Fig.S23 ^{1}H NMR spectrum of compound **4** (400 MHz, MeOH-d_4).

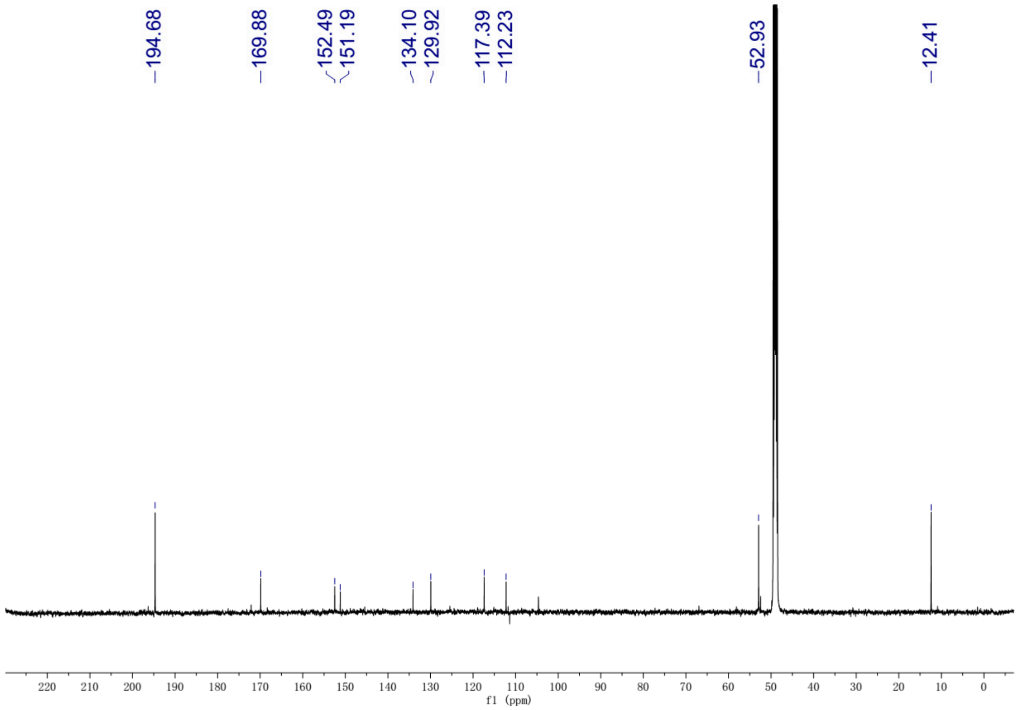

Fig.S24 ^{13}C NMR spectrum of compound **4** (100 MHz, MeOH-d_4).

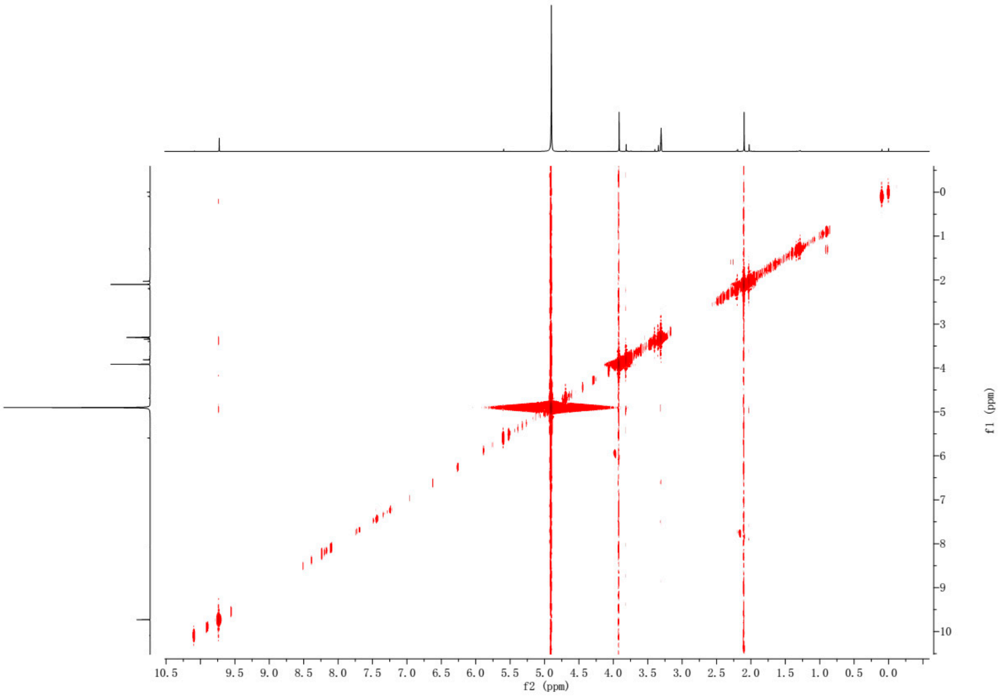

Fig.S25 ^{1}H-^{1}H COSY spectrum of compound **4** (MeOH-*d*$_4$).

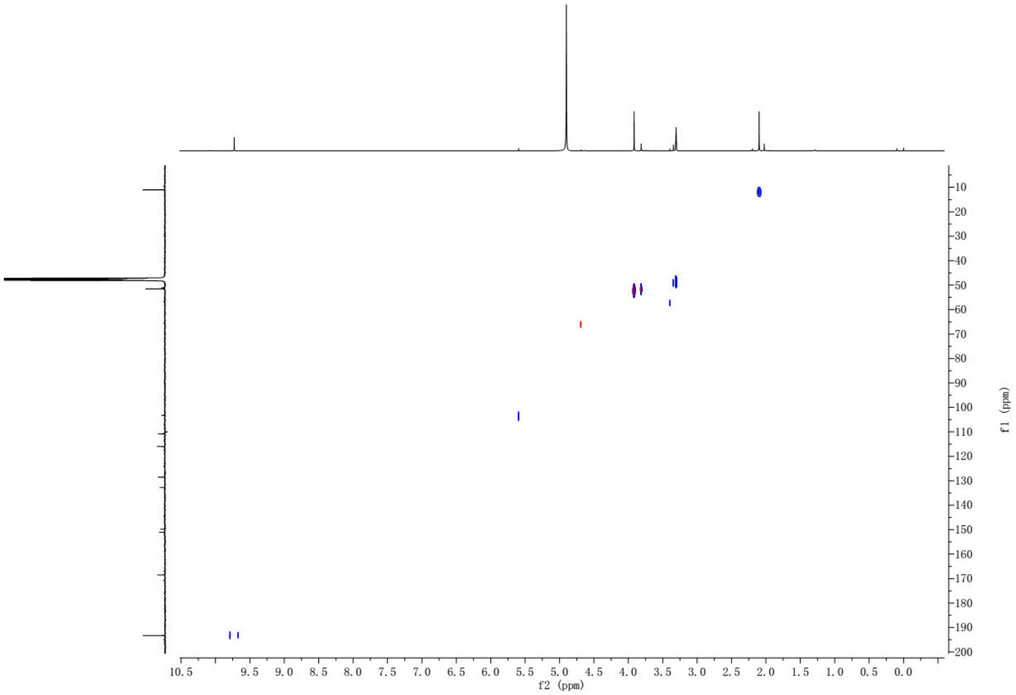

Fig.S26 HSQC spectrum of compound **4** (MeOH-*d*$_4$).

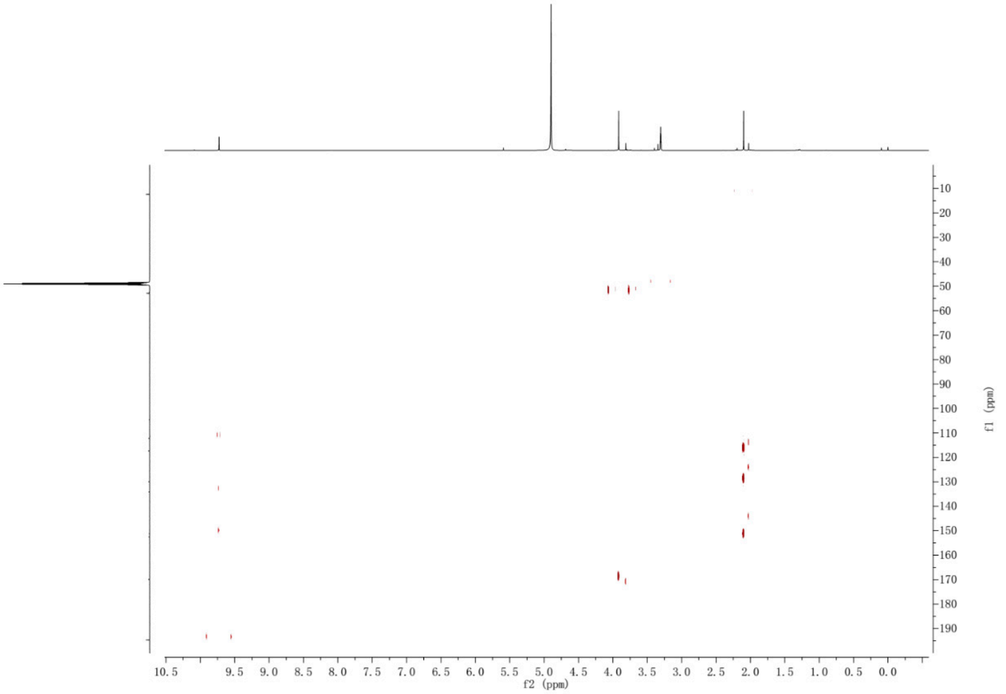

Fig.S27 HMBC spectrum of compound **4** (MeOH-d_4).

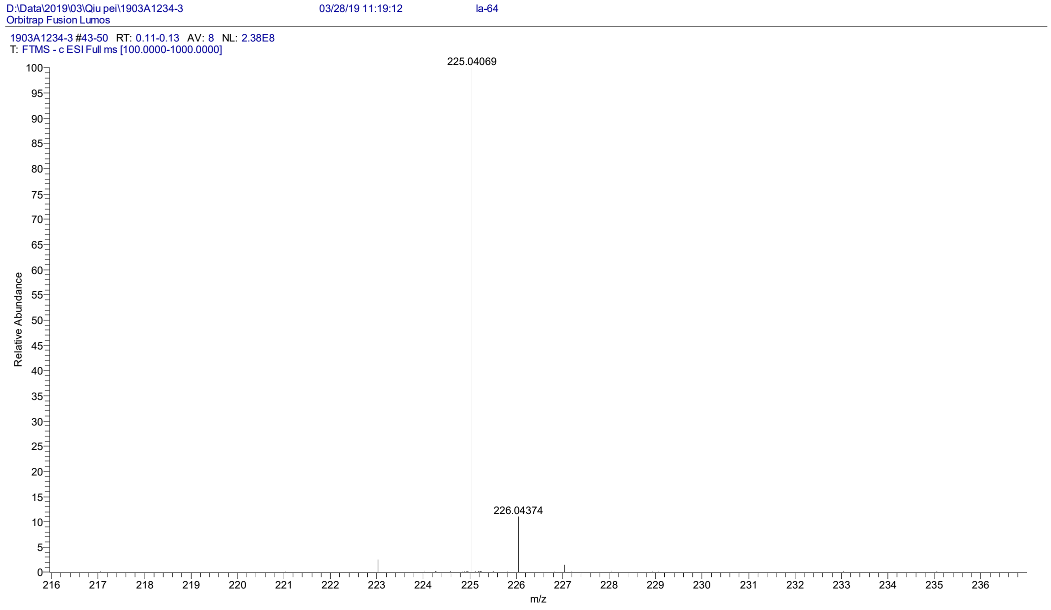

Fig.S28 HRESIMS spectrum of compound **4**.

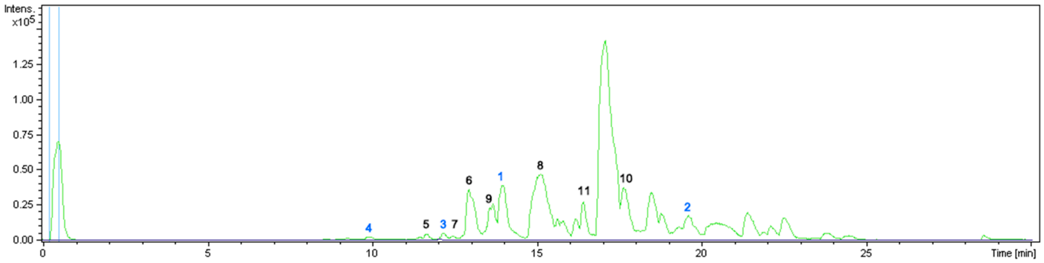

Retention time	Compounds	Mass [M+H]+	Calc. Mass	Element composition
9.8	4	227.0558	227.0550	$C_{10}H_{10}O_6$
11.7	5	197.0448	197.0444	$C_9H_8O_5$
12.1	3	181.0853	181.0859	$C_{10}H_{12}O_3$
12.4	7	317.1239	317.1231	$C_{14}H_{20}O_8$
12.9	6	197.0817	197.0808	$C_{10}H_{12}O_4$
13.5	9	977.5152	977.5158	$C_{57}H_{72}N_2O_{12}$
13.9	1	564.2861	564.2956	$C_{33}H_{41}NO_7$
15.1	8	359.0771	359.0761	$C_{18}H_{14}O_8$
16.4	11	348.0849	348.0857	$C_{17}H_{16}O_8$
17.7	10	418.2582	418.2588	$C_{24}H_{35}NO_5$
19.6	2	333.1337	333.1333	$C_{18}H_{20}O_6$

Fig.S29 The LC-HRESIMS analysis profile of crude extract.
